# Supplementary material for: Single-shot observation of optical rogue waves in integrable turbulence using time microscopy
Source: Nat Commun. 2016 Oct 7;7:13136. doi: 10.1038/ncomms13136 (PMC5059780; doi:10.1038/ncomms13136)
Supplement: Supplementary Information — Supplementary Figures 1-5, Supplementary Notes 1-3 and Supplementary References. [file ncomms13136-s1.pdf]

## SUPPLEMENTARY FIGURES

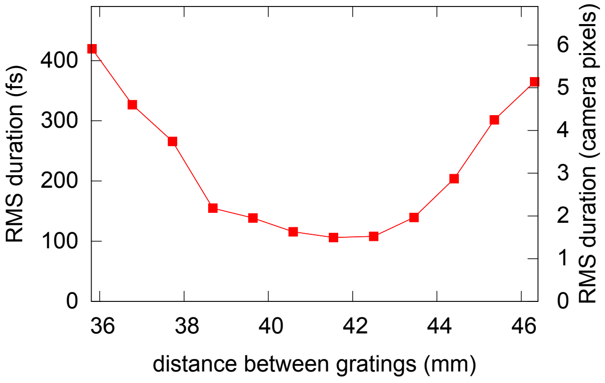


**Supplementary Figure** 1. Pulse duration measured with the time microscope, versus distance between grating planes in the 1560 nm compressor. Input pulses are produced by a 70 fs Erbium doped fiber laser. Each value has been determined by fitting a Gaussian function on the pulse shape. The minimum value corresponds to 250 fs FWHM.

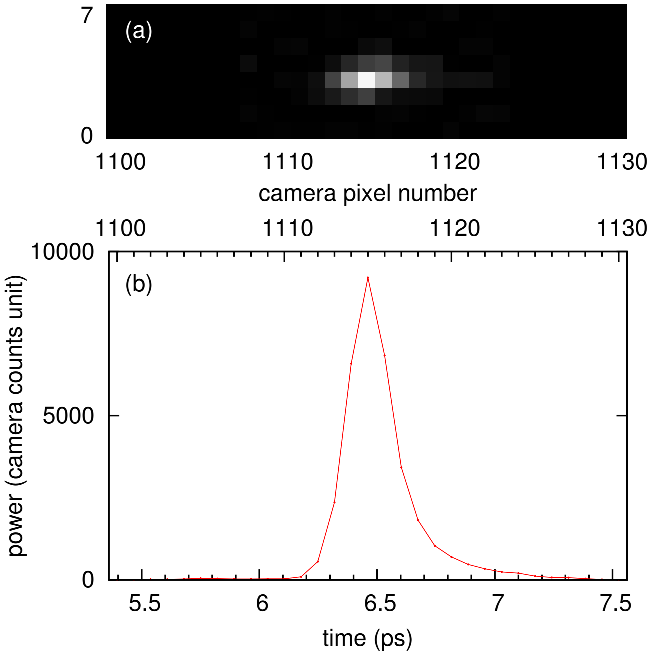


**Supplementary Figure** 2. Resolution of the time microscope, obtained by recording the response to a 70 fs pulse. (a) raw image recorded by the camera (the image size is 2048x8 pixels, but for clarity, 31x8 are represented). (b) Signal obtained from an horizontal cut of the upper image. The FWHM of the (b) curve –i.e. the time microscope resolution– is ≈ 250 fs FWHM.

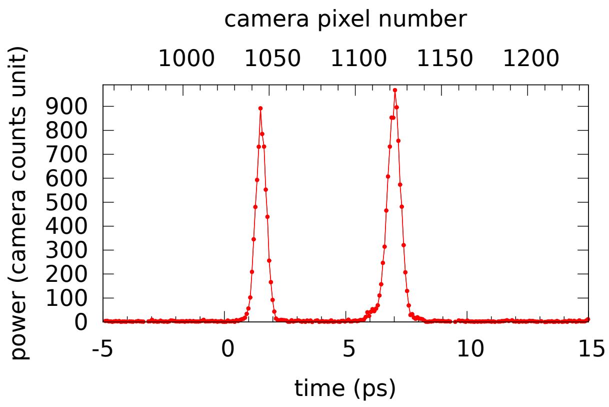


**Supplementary Figure** 3. Time microscope response to a series of two pulses (700 fs long) with known spacing (5.5 ps). This gives the correspondence between pixel spacing and time: 71 fs/pixel. Note that the lower scale has been defined after this calibration has been made.

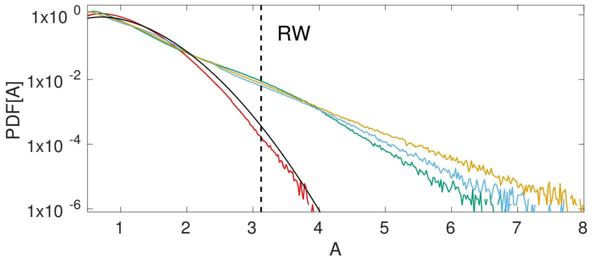


**Supplementary Figure** 4. Probability Density Function (PDF) of the amplitudes $A = \sqrt{P/\langle P \rangle}$ of the partially coherent light at the input of the fiber (red line) and at the output of the fiber for mean powers $\langle P \rangle = 500$ mW (green line), 2.6 W (blue line), 4 W (yellow line). The width of the initial optical spectrum is $\Delta\nu = 0.1$ THz. The black line represents the Rayleigh distribution [Eq. (1)]. The vertical dashed black line represents the calculated threshold of RWs computed for $\langle P \rangle = 4$W.

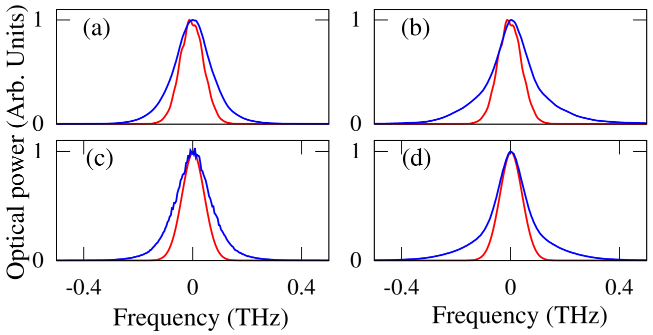


**Supplementary Figure** 5. Optical power spectra of the partially coherent light wave at the input (red lines) and output (blue lines) ends of the nonlinear fiber: (a), (b) experiments and (c), (d) numerical simulations of the 1D-NLSE. In (a), (c), $\Delta\nu = 0.1$ THz and $\langle P \rangle = 1$ W. In (b), (d), $\Delta\nu = 0.1$ THz and $\langle P \rangle = 2.6$ W.

## SUPPLEMENTARY NOTE 1. ADJUSTMENT AND CHARACTERIZATION OF THE ULTRAFAST RECORDING SETUP

### Temporal resolution: optimization and measurement

The temporal resolution of the time microscope is critically determined by the adjustment of the relative dispersions of the 1560 nm and 800 nm paths. We fixed the 800 nm dispersion (normal dispersion) to $\approx 20$ ps$^2$, by adjusting the grating compressor which is already present in our Titanium-Sapphire regenerative amplifier. This determined the temporal window of the analysis ($\approx 20$ ps here). Then we adjusted the dispersion of the 1560 nm compressor (which is anomalous, see Fig. 2 of the article) until best resolution was achieved. Conceptually, this corresponds to an adjustment of the focal time of the time lens (microscope objective in Fig. 2 of the article), until a focused image appears at the time microscope's output.

For this purpose, we injected short pulses ($\approx 70$ fs long) at 1560 nm, generated by an Erbium fiber laser at the time microscope input. Then we analyzed the time microscope data versus compressor dispersion. The pulse duration versus grating distance (perpendicular distance between grating planes) is represented in Supplementary Figure 1, and a typical response at best resolution is displayed in Supplementary Figure 2.

### Temporal calibration

The signal at the time microscope output consists of a linear image, which is recorded by the sCMOS camera (see Methods). In order to determine the correspondence between input time and pixel spacing, we proceeded as follows.

After performing the optimization described in the previous section, we produced a series of two pulses with known spacing. Technically, we injected linearly polarized 700 fs pulses in a polarization maintaining fiber, at 45 degrees with respect to the birefringent axes. By placing a polarizer at the fiber exit (also at 45 degrees with respect to the fiber's axes), series of two pulses were produced. We measured independently the pulse spacing (5.5 ps). Then, from the time microscope data obtained in these conditions (Supplementary Figure 3), we easily obtained the correspondence between pixel spacing and time. The scaling was 71 fs/pixel for all results presented in the article.

## SUPPLEMENTARY NOTE 2. PROBABILITY DENSITY FUNCTION FOR THE AMPLITUDE OF THE FIELD AND THRESHOLD OF ROGUE WAVES

We report here the probability density function (PDF) of the amplitude $A = \sqrt{P/\langle P\rangle}$ that is computed from the experimental data presented in the article. We also compute the power threshold characterizing rogue waves by using the usual definition commonly employed in hydrodynamics.

The data already used to compute the PDF of $P/\langle P\rangle$ shown in Fig. 3(a) of the article have been re-used to compute the PDF of $A$ together with the threshold of RWs. Let us first consider partially coherent waves having a spectral width $\Delta\nu = 0.1$THz that are emitted by the random light source and used as initial conditions in our experiments.

The PDF of $A$ of the initial wave is displayed in red on Supplementary Figure 4. It is shown in the article that the PDF of $P/\langle P\rangle$ is extremely close to the exponantial distribution : $\exp(P/\langle P\rangle)$. Considering a random process $x$ and a real function $y(x)$, the PDF of $y$ can be related to the PDF of $x$ through the simple relation : $PDF(y) = PDF(x)/|dy/dx|$ [1]. For an optical wave having a PDF of $P/\langle P\rangle$ that is given by the exponential distribution $\exp(P/\langle P\rangle)$, it can be easily shown that PDF of the amplitude $A = \sqrt{P/\langle P\rangle}$ reads [1, 2]:

$$PDF(A) = 2\sqrt{A}e^{-A^2} \qquad (1)$$

which is the Rayleigh distribution [2]. As stated in the article, the fact that the power $|\psi|^2$ has an exponential distribution can thus be directly associated to the fact that the amplitude $|\psi|$ has a Rayleigh distribution.

The Rayleigh distribution is plotted in black line in Supplementary Figure4. The PDF of the amplitude $A$ of the optical wave used as initial condition in our experiment is shown in red in Supplementary Figure 4, thus demonstrating that the statistics of the amplitude of our initial condition is very close to the Rayleigh's one.

Assuming a uniform distribution for the phase of $\psi$, the Rayleigh distribution of $A$ and the exponential distribution of $P$ correspond to a Gaussian statistics for the real and imaginary parts of $\psi$ (*i.e.* to a Gaussian statistics for the surface elevation $\eta$ in Hydrodynamics or for the electric field in Optics) [1, 2]. Note that the relationship between Gaussian, Rayleigh and exponential distributions is valid both in two [2] and one [1] dimensional systems. The comparison between the initial PDF of $A$ and the Rayleigh distribution confirms that random wave used as initial condition actually results from the linear superposition of waves and that the initial statistics indeed obeys the central limit theorem.

Supplementary Figure 4 also represents the PDF of the amplitudes $A$ measured at the output of the fiber for

mean powers of $\langle P \rangle = 0.5$W, $\langle P \rangle = 2.6$W and $\langle P \rangle = 4$W. Whatever the variable under consideration (amplitude here or power in the article), the heavy-tail of the statistics found at the output of the fiber becomes more and more pronounced when the mean power is increased.

In Oceanography, the significant wave height $H_S$ is often defined as representing the mean value of the height of the one-third highest waves [2]. The threshold defining Rogue Waves (RWs) height is often given by $H_{RW} = 2.2 H_S$ [2] so that waves having heights $H > H_{RW}$ are considered as being RWs.

The propagation of uni-directional deep water waves is described at the leading order by the 1D-NLSE (Eq. (1) of the article) that describes the nonlinear evolution of a complex envelope $\psi(z,t)$ [3, 4]. For water waves, the surface elevation $\eta$ is the real part of $\psi \exp i(k_0 z - \omega_0 t)$ where $\omega_0$ and $k_0$ are the mean pulsation and the mean wavenumber of the narrow band wave train under consideration [3]. In the limit of small bandwidth, the wave height $H$ (crest to trough) is given by the relation $H = 2|\psi|$ [2].

In analogy with hydrodynamics, the criterion defining RWs can be applied in optics to any variable that is proportionnal to $|\psi| = \sqrt{P}$. Here, we will consider the normalized amplitude $A$ defined as $A = \sqrt{P/\langle P \rangle}$.

The mean value of one-third of the highest values reached by $A$ is $A_S \sim 1.37$. Therefore the threshold of RWs is $A_{RW} = 2.2 \times A_S \sim 3$ which corresponds to a threshold in terms of optical power that reads $P_{RW}/\langle P \rangle \simeq 9$.

Considering the optical random wave at the output of the fiber at a mean power $\langle P \rangle = 4$W, the mean value of one-third of the highest values reached by $A$ is $A_S \sim 1.4$ and the threshold of RW is $A_{RW} \sim 3.1$ which corresponds to a power threshold $P_{RW}/\langle P \rangle \sim 9.7$. Note that the threshold of RWs defined with the one-third highest amplitudes is slightly lower before the nonlinear propagation ($A_{RW} \sim 3.$) than after the nonlinear propagation ($A_{RW} \sim 3.1$). The dashed line plotted in Supplementary Figure 4 represents the threshold computed in the highest nonlinear case after propagation in the fiber ($\langle P \rangle = 4$W).

## SUPPLEMENTARY NOTE 3. COMPARISON BETWEEN NUMERICAL SIMULATIONS AND EXPERIMENTS : OPTICAL SPECTRA

Starting from partially coherent waves with spectral width $\Delta\nu = 0.1$ THz, numerical simulations of the one-dimensional Nonlinear Schrödinger Equation (1D-NLSE) is performed. Supplementary Figure 5 displays the experimental and numerical spectra for two values of the mean power $\langle P \rangle$. For these set of parameters, the optical spectra observed in the experiments are quantitatively described by the 1D-NLSE. One observes the spectral broadening associated to the nonlinear propagation.

## SUPPLEMENTARY REFERENCES


[1] Goodman, J. W. *Statistical optics* (New York, Wiley-Interscience, 1985).

[2] Kharif, C., Pelinovsky, E. & Slunyaev, A. *Rogue waves in the ocean* (Springer, New York, 2009).

[3] Osborne, A. *Nonlinear ocean waves* (Academic Press, 2010).

[4] Chabchoub, A. *et al.* The nonlinear schrodinger equation and the propagation of weakly nonlinear waves in optical fibers and on the water surface. *Annals of Physics* **361**, 490 – 500 (2015).
